# MATTERING AND MOBILITY: POSITIVE RELATIONS WITH OTHERS PROTECTS AGAINST FUNCTIONAL DECLINE IN AGING ADULTS

**DOI:** 10.1093/geroni/igz038.2381

**Published:** 2019-11-08

**Authors:** Hannah Baker, Meghan Mitoraj, Elliot M Friedman

**Affiliations:** Purdue University, West Lafayette, Indiana, United States

## Abstract

Social relationships are robust predictors of better health and greater longevity in aging adults. The current study examined a relatively understudied aspect of relationship quality - positive relations with others, a domain of eudaimonic well-being - and its independent associations with change in and incidence of mobility limitations in a sample of mid-life and older adults from the Midlife in the United States (MIDUS) study. Using data from all 3 waves of MIDUS, we examined the extent to which positive relations with others predicted smaller increases in limitations and reduced risk of onset of new limitations over a 18-20 follow-up period. Linear and logistic regression models, adjusted for demographic and health confounds, showed that higher positive relations scores predicted slower increase in limitations over time (p=.003) and reduced risk of incidence of new limitations (p=.01). These effects were also observed over and above the associations with more traditional structural and functional measures of social relationships, neither of which was significantly linked to changes in functional abilities. These results suggest that positive relations with others may act as a unique protective factor for functional decline, both in individuals with no initial functional limitations and in those with existing limitations. They also extend prior research on the potential health benefits of social relationships to include a social dimension of eudaimonic well-being. Finally, they suggest that vulnerability to functional limitations and the potential benefits of social connectedness extend to mid-life as well as older adults.

